# Case Report: Long-term survival in FIGO stage IVB small cell carcinoma of the ovary, pulmonary type following complete cytoreduction and platinum re-challenge

**DOI:** 10.3389/fonc.2026.1964342

**Published:** 2026-10-19

**Authors:** Hyun Joo Chae, Youn Seok Choi, Jong Im Lee, Yoon Young Jeong

**Affiliations:** 1 Department of Obstetrics and Gynecology, School of Medicine, Daegu Catholic University, Daegu, Republic of Korea; 2 Department of Pathology, School of Medicine, Daegu Catholic University, Daegu, Republic of Korea

**Keywords:** case report, cytoreductive surgery, long-term survival, ovarian neuroendocrine carcinoma, platinum re-challenge, small cell carcinoma of the ovary, pulmonary type

## Abstract

**Background:**

Small cell carcinoma of the ovary, pulmonary type (SCCOPT), is a rare and highly aggressive malignancy with poor prognosis. Long-term survival in advanced-stage disease has rarely been reported.

**Case presentation:**

A 55-year-old postmenopausal woman presented with FIGO stage IVB SCCOPT accompanied by extensive peritoneal carcinomatosis and a solitary liver metastasis. No pulmonary lesion suggestive of primary lung malignancy was identified on CT, PET/CT, or chest radiography. She underwent aggressive primary cytoreductive surgery achieving complete macroscopic resection (R0), followed by four cycles of cisplatin and etoposide (EP). Sixteen months after diagnosis, isolated intracranial recurrence with multiple brain metastases were successfully treated with radiotherapy. Twenty-one months after diagnosis, systemic recurrence involving multiple liver metastases and a spinal metastasis was treated with six cycles of EP re-challenge, resulting in a marked radiologic response and subsequent durable disease control. The patient remained alive without clinical evidence of disease progression at the latest follow-up, 104 months after diagnosis.

**Conclusions:**

This case suggests that prolonged survival may be achievable in selected patients with advanced SCCOPT through aggressive multimodal treatment. Complete cytoreductive surgery, platinum-based chemotherapy, including EP re-challenge following a prolonged platinum-free interval and previous response to platinum-based chemotherapy, and timely local treatment of metastatic lesions may contribute to durable disease control.

## 1 Introduction

Primary small cell carcinoma of the ovary is a rare and highly aggressive neoplasm, accounting for less than 1% of all ovarian malignancies (1). Two distinct clinicopathologic entities are recognized in the 2020 WHO classification: small cell carcinoma of the ovary, hypercalcemic type (SCCOHT), and pulmonary type (SCCOPT) (2).

SCCOPT predominantly affects perimenopausal or postmenopausal women, is not associated with hypercalcemia, and shares morphologic and immunohistochemical features with small cell lung cancer (SCLC) (3, 4). It is classified as a high-grade neuroendocrine carcinoma and typically expresses neuroendocrine markers including CD56, synaptophysin, and chromogranin (4, 5). Given its rarity, no consensus treatment guidelines exist, and management is largely extrapolated from SCLC protocols, most commonly platinum-based chemotherapy combined with etoposide (3, 4).

SCCOPT carries a poor prognosis even when diagnosed at an early stage, and long-term survival in advanced-stage disease, particularly FIGO stage IV, has rarely been reported (5–8). Herein, we report a case of stage IVB SCCOPT presenting with extensive peritoneal carcinomatosis and a solitary, resectable liver parenchymal metastasis, in which aggressive cytoreductive surgery and platinum-based chemotherapy, including re-challenge at recurrence, resulted in prolonged survival exceeding eight years.

## 2 Case description

### 2.1 Diagnostic assessment and primary treatment

In July 2017, a 55-year-old postmenopausal woman (gravida 3, para 3) presented with a one-month history of abdominal distension, dyspepsia, and constipation. Her past medical history was unremarkable. A Papanicolaou smear performed in January 2017 and both esophagogastroduodenoscopy and colonoscopy performed in June 2017 were normal. Laboratory evaluation revealed a normal serum calcium level of 9.0 mg/dL; tumor markers were elevated, with cancer antigen 125 (CA-125) at 689 U/mL and CA 19–9 at 40 U/mL.

Preoperative CT and PET/CT demonstrated a large hypermetabolic pelvic mass (maximum standardized uptake value [SUVmax] 11.28), extensive peritoneal and omental carcinomatosis with ascites (SUVmax 12.68), a solitary liver metastasis, and multiple enlarged aortocaval, para-aortic, and mesenteric lymph nodes (SUVmax 7.56). No pulmonary lesion suggestive of a primary lung malignancy was identified on CT, PET/CT, or chest radiography. Preoperative imaging suggested an ovarian primary with a solitary, resectable liver parenchymal metastasis, supporting an aggressive surgical approach; thus, an extensive primary cytoreductive surgery was performed, achieving complete macroscopic resection (R0). The overall clinical course, including treatment, recurrence, and long-term follow-up, is summarized in **Table 1**.

**Table 1:** Clinical course, interventions, and outcomes according to time from diagnosis.

Time from diagnosis	Clinical course	Intervention/Outcome
At diagnosis	FIGO stage IVB SCCOPT with peritoneal carcinomatosis and liver metastasis	Primary cytoreductive surgery with complete macroscopic resection (R0)
Postoperative period	Histopathologic diagnosis of SCCOPT	Etoposide plus cisplatin (EP), 4 cycles
16 months	Brain metastasis	Brain radiotherapy
21 months	Systemic recurrence involving the liver and vertebra	EP re-challenge, 6 cycles
After EP re-challenge	Marked radiologic response with durable disease control	Surveillance
104 months	Long-term follow-up	Alive without clinical evidence of recurrent or progressive disease

FIGO, International Federation of Gynecology and Obstetrics; SCCOPT, small cell carcinoma of the ovary, pulmonary type; R0, complete macroscopic resection with no gross residual disease; EP, etoposide plus cisplatin.

Intraoperative findings demonstrated extensive tumor involvement of the pelvic cavity, including the uterus, bilateral adnexa, and rectum, with diffuse peritoneal carcinomatosis throughout the abdominal cavity. Metastatic deposits were identified on both hemidiaphragms, both paracolic gutters, and the mesenteric surface. Enlarged para-aortic lymph nodes were also noted. The following procedures were performed: hysterectomy with bilateral salpingo-oophorectomy, pelvic peritonectomy, bilateral paracolic gutter peritonectomy, bladder peritonectomy, bilateral diaphragmatic peritonectomy, total omentectomy, bilateral pelvic and para-aortic lymph node dissection, excision of mesenteric surface implants, low anterior resection, and liver wedge resection. Complete macroscopic resection was achieved with no gross residual disease. The operative time was approximately 13 hours, with an estimated blood loss of 2, 000 mL. The postoperative course was uneventful, without significant complications, and the patient was able to initiate the first cycle of adjuvant chemotherapy approximately 3 weeks after surgery.

Histopathological examination of the bilateral ovaries (right: 8.0 × 6.0 × 5.0 cm; left: 3.5 × 2.5 × 1.0 cm) demonstrated bilateral involvement by small cell neuroendocrine carcinoma, with a dominant tumor in the right ovary. The tumors were composed of diffuse sheets of highly atypical tumor cells. Tumor cells showed hyperchromatic nuclei, scanty cytoplasm and abundant mitotic activity. No associated epithelial or germ-cell tumor component was identified. All resected specimens suspected of metastasis, including liver, large intestine, omentum, the para-aortic lymph nodes (5 of 6 positive), showed identical histology, confirming widespread metastatic disease. Immunohistochemical staining showed positivity for cytokeratin, CD56, chromogranin, TTF-1, p16, p53 with negativity for vimentin and WT-1, indicating neuroendocrine differentiation. The Ki-67 proliferation index was 74.8%. Additionally, SMARCA4 expression was retained in the tumor cells. Based on these clinicopathologic findings, a diagnosis of primary small cell neuroendocrine carcinoma of the ovary, pulmonary type, FIGO stage IVB, was established (**Figure 1**). Postoperatively, the patient received four planned cycles of adjuvant chemotherapy with cisplatin (70 mg/m², day 1) and etoposide (100 mg/m², days 1–3).

**Figure 1:**
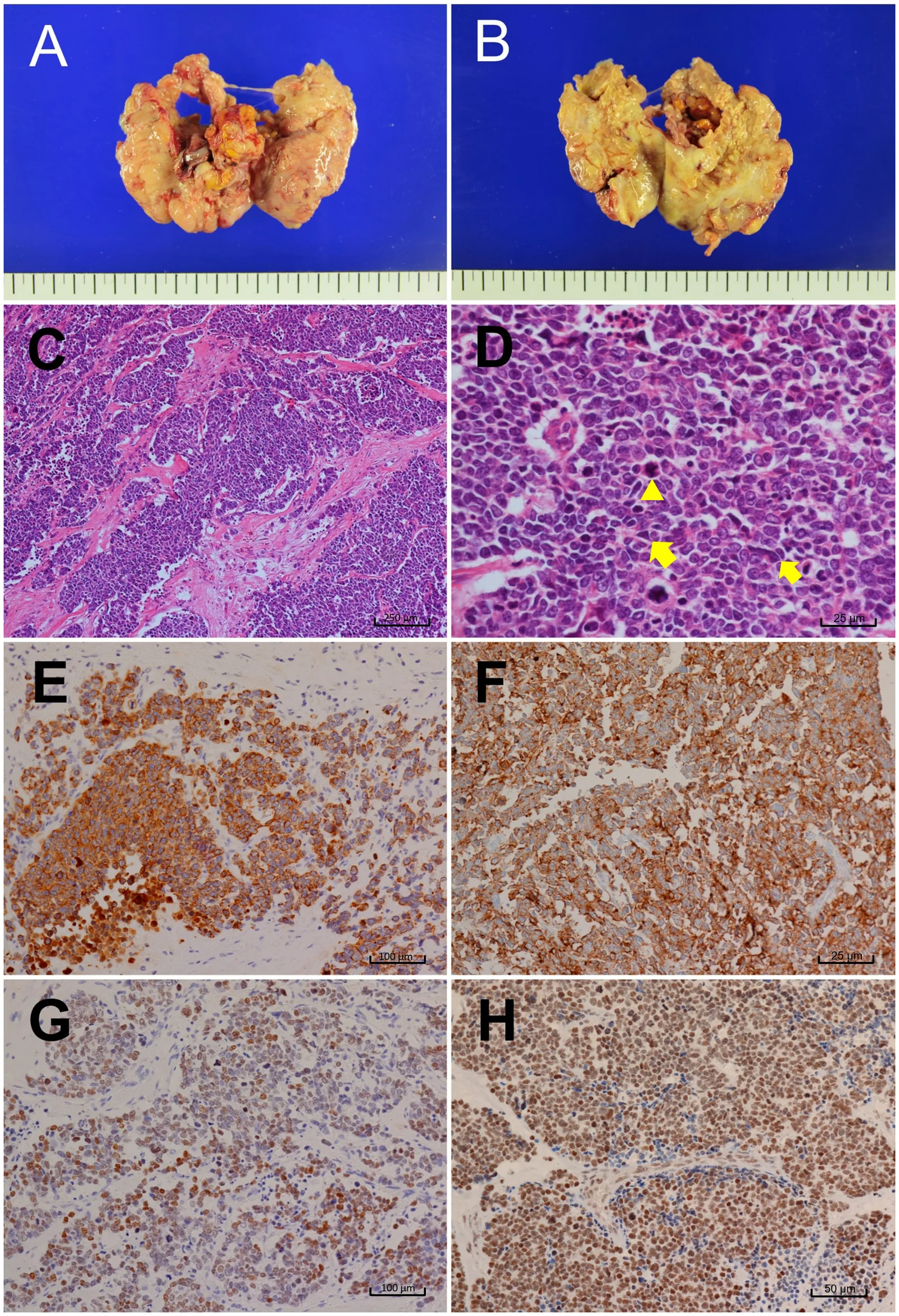
Gross, histopathologic, and immunohistochemical findings of small cell carcinoma of the ovary, pulmonary type. **(A, B)** Gross examination of the ovarian mass showing a solid tumor with irregular cut surfaces and focal hemorrhagic and necrotic changes. **(C)** Low-power hematoxylin and eosin (H&E) image demonstrating large sheets and clusters of small blue round tumor cells with scant cytoplasm. **(D)** High-power H&E image revealing nuclear molding (arrows), finely dispersed chromatin, and frequent mitotic figures (arrowhead). **(E–H)** Immunohistochemical staining demonstrating positivity for cytokeratin **(E)**, chromogranin **(F)**, TTF-1 **(G)**, and retained SMARCA4 expression **(H)**.

### 2.2 Recurrence and therapeutic intervention

Sixteen months after diagnosis, she developed dizziness, nausea, and vomiting. Brain MRI revealed four metastatic lesions involving the bilateral cerebellar hemispheres, cerebellar vermis, and right parietal lobe, without extracranial recurrence. She underwent whole-brain radiotherapy (30 Gy in 10 fractions) followed by a cerebellar boost (9 Gy in 5 fractions) using 3D-CRT. Follow-up MRI showed a radiographic partial response, with further reduction in lesion size on subsequent imaging. Twenty-one months after diagnosis, PET/CT demonstrated systemic recurrence with multiple hepatic metastases and an L1 vertebral metastatic lesion. Re-challenge chemotherapy with 6 cycles of cisplatin and etoposide was administered. Post-treatment abdominopelvic CT demonstrated a marked radiologic response of the hepatic metastases, with only a small residual hypodense hepatic lesion remaining. On subsequent follow-up imaging, this residual lesion further decreased in size without evidence of progression (**Figure 2**).

**Figure 2:**
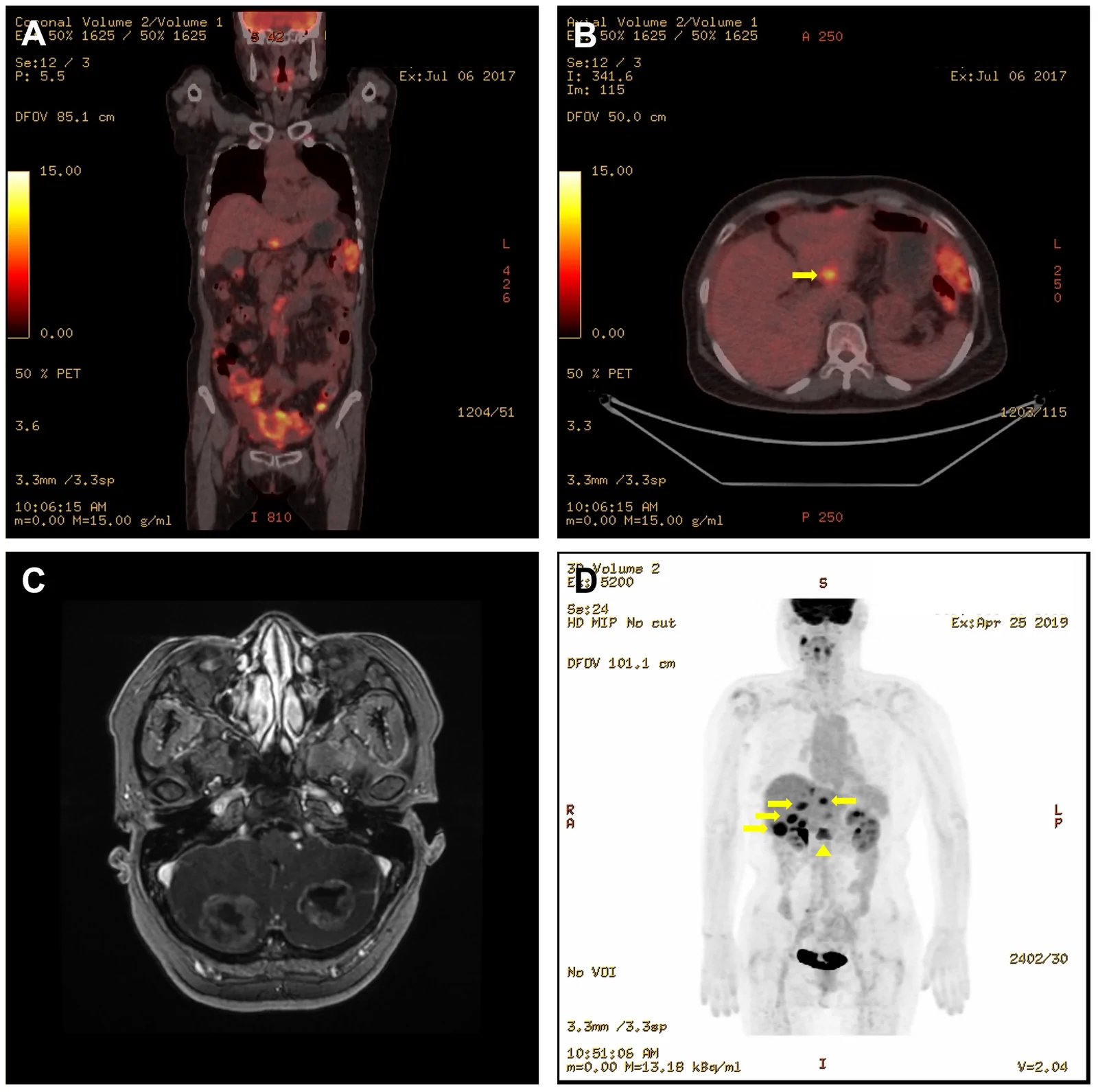
Radiologic findings and metastatic progression of pulmonary-type ovarian small cell carcinoma. **(A)** Initial fluorodeoxyglucose positron emission tomography/computed tomography (FDG PET/CT) demonstrating hypermetabolic lesions involving the abdomen and pelvis at the time of diagnosis. **(B)** Axial FDG PET/CT image showing focal hypermetabolic uptake within the liver parenchyma (arrow), suspicious for hepatic metastasis. **(C)** Brain magnetic resonance imaging (MRI) demonstrating a metastatic lesion in the cerebellum. **(D)** Follow-up FDG PET/CT demonstrating multiple hypermetabolic lesions involving the liver (arrows) and vertebra (arrowhead), suggestive of metastatic progression.

### 2.3 Follow-up and outcomes

Long-term surveillance imaging at approximately 83–84 months after diagnosis, including whole-body bone scan, chest CT, abdominopelvic CT, and PET/CT, confirmed a sustained treatment response. Previously identified liver lesions had nearly completely resolved, and the L1 vertebral lesion showed a reduction in size and metabolic activity, with no evidence of active intrathoracic or abdominopelvic disease. Follow-up imaging at approximately 88 months after diagnosis confirmed stable disease without interval progression; brain MRI demonstrated focal tissue loss and mild leukoencephalopathy consistent with treatment-related changes, without evidence of acute pathology. This was the most recent radiologic assessment, and no additional imaging was performed thereafter. At the most recent clinical follow-up, approximately 104 months after diagnosis, the patient remained alive without clinical evidence of recurrent or progressive disease.

## 3 Discussion

Primary small cell carcinoma of the ovary, pulmonary type (SCCOPT), is an exceptionally rare and highly aggressive malignancy with a poor prognosis. Most patients present with advanced-stage disease and experience rapid progression despite multimodal treatment, with a median overall survival of 14 months and death within 2 years in the majority of cases (3, 4, 9). Because of its rarity and morphologic overlap with other entities, accurate diagnosis requires careful clinicopathologic correlation to distinguish SCCOPT from small cell carcinoma of the ovary, hypercalcemic type (SCCOHT), and metastatic small cell lung carcinoma (SCLC) (3, 4).

In the present case, the diagnosis of SCCOPT was supported by both clinical and pathologic findings. SCCOHT was considered in the differential diagnosis; however, it typically occurs in younger women and is frequently associated with hypercalcemia and loss of SMARCA4(BRG1) expression. In contrast, our patient was a 55-year-old postmenopausal woman with a normal serum calcium level, and SMARCA4 expression was retained in the tumor cells.

The tumor also demonstrated WT-1 negativity, diffuse mutant-type p53 overexpression, and positivity for neuroendocrine markers including CD56 and focal chromogranin, further supporting SCCOPT over SCCOHT. Although TTF-1 positivity raised the possibility of metastatic SCLC, TTF-1 expression alone is not specific for pulmonary origin. No primary pulmonary lesion was identified on CT, PET/CT, or chest radiography at diagnosis, and long-term surveillance imaging continued to show no evidence of a pulmonary primary or active intrathoracic disease. Together with the ovarian disease distribution and pathologic findings, these findings favored primary ovarian SCCOPT over metastatic SCLC.

Given the absence of standardized treatment guidelines, management of SCCOPT is largely extrapolated from SCLC-based chemotherapy and surgical principles established for high-grade ovarian malignancies (3, 4, 10). Although direct evidence supporting the application of epithelial ovarian cancer surgical principles to SCCOPT remains limited (4), definitive histologic diagnosis is often unavailable before surgery in real-world practice. In such settings, surgical decision-making is commonly guided by a preoperative working diagnosis of epithelial ovarian cancer. In epithelial ovarian cancer, maximal cytoreductive surgery achieving complete macroscopic resection (R0) is a well-established prognostic factor (10). On this basis, aggressive cytoreduction may be a reasonable strategy in selected patients with SCCOPT when R0 resection is considered feasible.

The role of lymphadenectomy in SCCOPT remains unclear. In epithelial ovarian cancer, routine lymphadenectomy does not improve survival in the absence of suspicious pelvic or para-aortic lymph node enlargement (11), whereas resection of clinically suspicious enlarged nodes is performed as part of debulking surgery. Although a retrospective SEER-based study reported improved survival in patients with small cell carcinoma of the ovary who underwent surgery including lymphadenectomy (12), the limitations inherent to retrospective analyses preclude definitive conclusions. In the present case, para-aortic lymph node enlargement was identified, and lymph node dissection was performed as part of debulking surgery to achieve R0 resection.

Notably, aggressive debulking surgery was performed despite stage IV disease because the parenchymal liver metastasis was limited and amenable to complete resection by liver wedge resection. Although such an approach cannot be uniformly recommended for all patients with stage IV SCCOPT, the present case suggests that FIGO stage IVB disease alone should not necessarily preclude cytoreductive surgery when complete macroscopic resection is considered achievable. To better contextualize the present case, previously reported cases of FIGO stage IV SCCOPT are summarized in **Table 2**. Most reported patients had poor outcomes despite multimodal treatment, whereas prolonged survival has been exceptionally rare. Compared with previously reported cases, our patient achieved one of the longest documented survivals following complete macroscopic cytoreduction and subsequent successful platinum-based chemotherapy re-challenge.

**Table 2:** Published cases of FIGO stage IV small cell carcinoma of the ovary, pulmonary type (SCCOPT): treatment and clinical outcomes.

Author	Age	Metastasis	Operation	Adjuvant therapy	Survival
Reckova M et al. (2010)	67	Bone, bilateral breast	TAH, BSO, AP, partial omentectomy with multiple excision from peritoneal surface	Carboplatin + etoposide (6) Cisplatin + topotecan (1) Spine RT Extracerebral RT Palliative RT	DOD, 24 months
Gupta P et al. (2021)	60	NR	bilateral ovarian mass resection	Paclitaxel + Carboplatin (6)	Lost to follow-up after 1 month
	65	NR	TAH, BSO, bilateral retroperitoneal lymph node dissection	none	AWD, 2 months
	44	NR	TAH, BSO, OMT	Cisplatin + etoposide (6)	ANED, 12 months
	60	NR	BSO, omentectomy	Paclitaxel + Carboplatin (6)	AWD, 30 months
Junjun Li et al. (2025)	45	NR	TAH, BSO, LAR, LYM	none	DOD, 0.9 months
	67	NR	TAH, BSO, OMT, LYM, AP, S2H, PIR	Cisplatin + etoposide (6)	DOD, 12 months
Present case	55	Liver and para-aortic/aortocaval lymph nodes	TAH, BSO, extensive peritonectomy, omentectomy, bilateral pelvic and para-aortic lymphadenectomy, LAR, liver wedge resection	Cisplatin + etoposide (4) Cisplatin + etoposide (6) Brain RT	ANED, 104 months

NR, Not reported; TAH, total abdominal hysterectomy; BSO, bilateral salpingo-oophorectomy; OMT, omentectomy; AP, appendectomy; LYM, lymphadenectomy; LAR, low anterior resection; PIR, pelvic implant resection; S2H, segment II hepatectomy; DOD, Dead of disease; AWD, Alive with recurrent or residual disease; ANED, Alive with no evidence of disease.

Published data indicate a high risk of recurrence in SCCOPT, with recurrence reported in approximately 60% of patients (18/30), most commonly involving the liver, brain, and bone (13). Evidence regarding treatment at recurrence is particularly limited, and the role of platinum re-challenge remains unclear (14).

Although the optimal chemotherapy regimen for SCCOPT remains undefined, cisplatin–etoposide is commonly used based on clinical experience in SCLC (3, 4). The post-recurrence course in this case is particularly noteworthy. In the present case, whole-brain radiotherapy (30 Gy in 10 fractions) was administered for multiple intracranial metastases, followed by a cerebellar boost (9 Gy in 5 fractions) to deliver an additional radiation dose to the cerebellar region containing multiple metastatic lesions (15).

In the present case, multiple hepatic metastases developed after a prolonged platinum-free interval of approximately 18 months following the last administration of cisplatin during first-line treatment. Given the previous response to EP and the prolonged platinum-free interval, the same regimen was re-administered and resulted in a marked radiologic response, with only a small residual hypodense hepatic lesion on post-treatment imaging that further decreased in size during subsequent follow-up. Remarkably, the patient subsequently achieved durable disease control, remaining alive for approximately 83 months after systemic recurrence.

The durable response to cisplatin–etoposide re-challenge following a prolonged platinum-free interval and a previous response to platinum-based chemotherapy suggests that these factors may be clinically relevant when considering platinum re-challenge in recurrent SCCOPT. Although no definitive conclusions can be drawn from a single case, the present experience may provide clinically relevant insight into potential treatment strategies for this rare malignancy.

A limitation of this case report is the lack of comprehensive molecular characterization. Next-generation sequencing, RB1 status, tumor mutational burden, and germline testing were not performed because such testing was not routinely implemented at our institution at the time of diagnosis in 2017. Therefore, potential molecular features associated with the exceptionally favorable clinical course could not be evaluated. In addition, given the nature of a single case, the contribution of complete cytoreduction and platinum re-challenge to long-term survival cannot be established, and favorable tumor biology may have substantially contributed to the prolonged survival observed in this patient.

## 4 Conclusion

This case suggests that prolonged survival may be achievable in selected patients with advanced SCCOPT through aggressive multimodal treatment. Even in FIGO stage IVB disease, complete cytoreduction may be considered in carefully selected patients when R0 resection appears feasible. Furthermore, the durable response to cisplatin–etoposide re-challenge following a prolonged platinum-free interval and previous response to platinum-based chemotherapy suggests that these factors may be clinically relevant when considering treatment options for recurrent SCCOPT.

## Data Availability

The raw data supporting the conclusions of this article will be made available by the authors, without undue reservation.
